# Postoperative Cervical Haematoma Complicated by Ipsilateral Carotid Thrombosis and Aphasia after Anterior Cervical Fusion: A Case Report

**DOI:** 10.1155/2013/590639

**Published:** 2013-03-07

**Authors:** Kingsley R. Chin, Jason Seale, Veronica Butron, Vanessa Cumming

**Affiliations:** ^1^Charles E. Schmidt College of Medicine, Florida Atlantic University and Institute for Modern and Innovative Surgery (iMIS), 1100 W. Oakland Park Boulevard, Suite No. 3, Fort Lauderdale, FL 33311, USA; ^2^iMIS Surgery, 1100 W. Oakland Park Boulevard, Suite No. 3, Fort Lauderdale, FL 33311, USA; ^3^Less Exposure Surgery (LES) Society, 300 E. Oakland Park Boulevard, Suite 502, Fort Lauderdale, FL 33334, USA

## Abstract

Hematoma alone is the most common vascular complication reported after anterior cervical decompression and fusion (ACDF). We present this case to report the occurrence of postoperative cervical hematoma complicated by ipsilateral carotid thrombosis and aphasia after an uncomplicated C4–6 ACDF. This is a case of a 65-year-old woman who underwent revision fusions of the C4-5 and C6-7 levels complicated by postoperative cervical hematoma and carotid thrombosis. The patient's history, clinical examination, imaging findings, and treatment are reported. The revision fusions were performed and deemed routine. Approximately eight hours later 200 mL of blood was evacuated from a postoperative cervical hematoma. The patient became unresponsive and disoriented a few hours after evacuating the hematoma. Computed tomography and magnetic resonance imaging of the brain were normal, but magnetic resonance angiography demonstrated total occlusion of the left carotid artery. Thrombectomy was performed and the patient was discharged without residual deficits. At the latest followup she is fully functional and asymptomatic in her neck. We suggest, after evacuating a cervical hematoma, an evaluation of the carotids be made with MRA or cerebral angiography, as this may demonstrate a clot before the patient develops symptoms.

## 1. Introduction

Anterior cervical decompression and fusion (ACDF), a common treatment for cervical disc disease, is associated with good outcomes and low complication rates [1–5]. Complications can be devastating, especially hematoma, vascular injury, esophageal injury, neurological deficits, or graft dislodgement [4, 6–9]. Complications related to the carotid artery during ACDF are rare [10], and thrombosis has never been reported in association with a postoperative cervical hematoma, although interruption of laminar blood flow during retraction is documented [11].

We report a case of postoperative cervical hematoma complicated by ipsilateral carotid thrombosis and aphasia after a revision ACDF at C4-5 and C6-7 for adjacent segment disease. This case is presented to share the first documented case including this series of complications and to be instructive in sharing our management experience.

## 2. Case Presentation

A 65-year-old female patient with a body mass index of 19.2 kg/m^2^ and past medical history including hepatitis C treated with interferon, Lyme disease, hypertension, osteoarthritis, lumbar laminectomy and fusion, C5-6 fusion, hysterectomy, and breast biopsy presented with multilevel spondylosis and adjacent level breakdown at C4-5 and C6-7. 

She underwent revision fusions of C4-5 and C6-7 levels with interbody PEEK cage (Invibio PEEK Optima), demineralized bone matrix, and cervical plates (SpineFrontier Indus InVue cervical plate, Beverly, MA, USA). These procedures were completed via a left-sided approach. Hemostasis was achieved before closing the wound. This procedure was completed and was deemed routine without surgical, anesthetic, or cord monitoring complications. 

Approximately 8 hours later swelling of the anterior neck was noted. This was assessed as a hematoma of the cervical spine causing airway compromise. Immediately the patient was returned to the operating suite for an urgent evacuation of the hematoma. Approximately 200 mL clotted blood was drained. Active oozing was noted from the muscles and cauterized. The wound was irrigated without force, then collagen sponge and gelatin matrix hemostatic sealant around the muscle areas were placed. After Penrose drain replacement and wound closure, excessive bleeding was noted, so the wound was reopened and more collagen sponge and thrombin were used along with cauterization. A bulb drain was placed with a 1/4 inch Penrose and the wound was closed.

**Figure 1:**
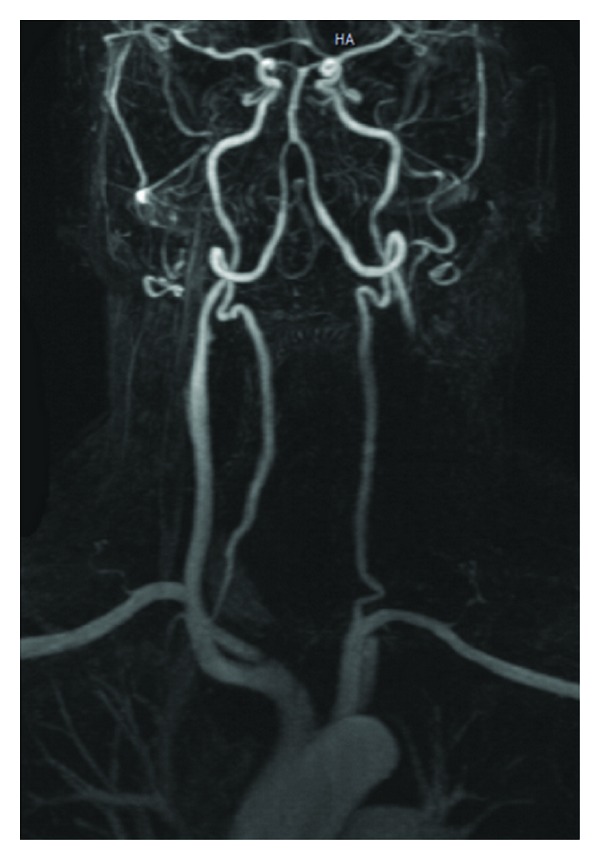
MRA postoperative day 2 demonstrates total occlusion of the left common carotid artery, including bifurcation and external carotid artery.

Later that evening, the patient became increasingly disoriented and eventually unresponsive to commands. Clinically posterior fossa dysfunction was assessed with the patient obtunded and eyes gazing downward. The possibility of a cerebrovascular accident (CVA) in the posterior fossa was considered and computed tomography (CT) brain ordered. CT brain was normal and MRI demonstrated no acute infarct; however magnetic resonance angiography (MRA) revealed total occlusion of the left common carotid artery, including the bifurcation and external carotid artery with some reconstitution of the internal carotid at the level of the siphon from collateral blood flow (Figure 1). Therefore, without delay, vascular surgeons performed exploration and thrombectomy of the left carotid artery. The vascular surgeon commented only on the large size of the thrombus. There was no obvious intimal damage or arteriosclerosis as reported postoperatively. After all vascular clamps were released and good pulsations obtained in the entire common, external, and internal carotid arteries, the heparin injected prior to clamping was reversed with protamine sulfate and hemostasis was considered satisfactory. Another Penrose drain was left in the surgical bed. 

**Figure 2:**
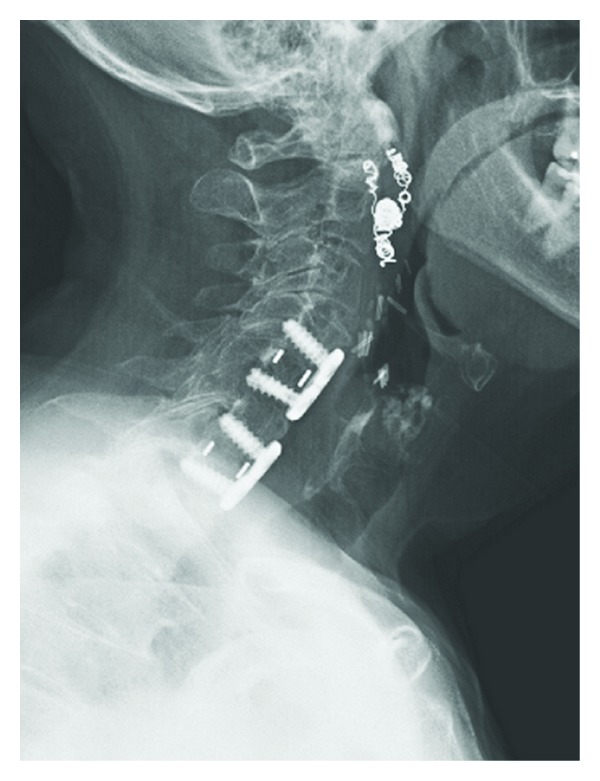
Postoperative lateral radiograph showing fixation at 9 months. (Please note: clips and coils postembolization of the occipital and external carotid arteries apparent in the anterolateral left neck.)

Our patient's hospital stay was further complicated by an acute right brachial deep vein thrombosis secondary to a line *in situ*, and a heparin-induced thrombocytopenia. She also suffered a reactive leucocytosis immediately postoperatively and a nosocomial (MSSE) pneumonia. However, the patient was discharged from hospital on postoperative day 20 without any residual deficits, and at her latest followup at nine months she is fully functional and asymptomatic in her neck. Of note: our patient, since these reported procedures, had occipital and external carotid artery embolization performed, the coils and clips are obvious on X-ray (Figure 2). These procedures were completed by a separate team of vascular surgeons for discrete indications and are not directly related to the reported events herewith.

## 3. Discussion 

Virchow's triad describes three broad categories which contribute to the development of thrombosis: stasis, endothelial damage, and/or hypercoagulability. In this case our patient was not in a hypercoagulable state as evidenced by the hematoma and her normal liver function and bleeding indices, despite a history of hepatitis C [12]. No endothelial damage was identified at thrombectomy in this case, and considering that her aphasia developed after removal of the hematoma, it is our suspicion that the hematoma compressed the carotid artery enough to decrease laminar blood flow inducing stasis and providing a nidus for the development of a thrombus. Chronic long-term occlusion of the carotid may be another explanation of this patient's second event postoperatively certainly, but her medical history yielded no prior report of CVA-type events. This may have been masked, however, by compensatory mechanisms such as elevated oxygen extraction fraction improvement and/or improvements in blood flow with chronic occlusion [13]. 

It is unknown whether she would have developed aphasia from compression within the same time frame had we not removed the hematoma; nonetheless, our experience should raise awareness and prompt prophylactic action before evacuating a hematoma. 

## 4. Conclusion

Our literature review yielded no prior cases detailing similar complications. This case documents perhaps the first report of this occurrence, is instructive, and raises awareness. We suggest after evacuating a cervical hematoma, especially in patients with risk factors, an evaluation of the carotids be made with MRA, Doppler ultrasonography, intraoperative pulse examination, or cerebral angiography as this may demonstrate a clot before the patient develops symptoms.
